# PACAP and VIP Neuropeptides’ and Receptors’ Effects on Appetite, Satiety and Metabolism

**DOI:** 10.3390/biology12071013

**Published:** 2023-07-17

**Authors:** John P. Vu, Leon Luong, Daniel Sanford, Suwan Oh, Alma Kuc, Rita Pisegna, Michael Lewis, Joseph R. Pisegna, Patrizia M. Germano

**Affiliations:** 1 Research Service, Veterans Affairs Greater Los Angeles Healthcare System, Los Angeles, CA 90073, USA; jvu@scorpiontx.com (J.P.V.); alma.kuc@va.gov (A.K.);; 2 CURE/Digestive Diseases Research Center, Department of Medicine, University of California, Los Angeles, CA 90073, USA; 3 Division of Hematology and Oncology, Department of Medicine, Cedars-Sinai Medical Center, Los Angeles, CA 90078, USA; michael.lewis8@va.gov; 4 Department of Pathology, Cedars-Sinai Medical Center, Los Angeles, CA 90048, USA; 5 Department of Pathology, Veterans Affairs Greater Los Angeles Health Care System, Los Angeles, CA 90073, USA; 6 Division of Gastroenterology, Hepatology and Parenteral Nutrition, VA Greater Los Angeles Healthcare System and Department of Medicine, Los Angeles, CA 90073, USA; 7 Division of Human Genetics, David Geffen School of Medicine at UCLA, Los Angeles, CA 90095, USA; 8 Division of Pulmonary and Critical Care, Veterans Affairs Greater Los Angeles Healthcare System, Los Angeles, CA 90073, USA

**Keywords:** PACAP, VIP, neuropeptides, metabolism, energy homeostasis

## Abstract

**Simple Summary:**

PACAP and VIP are peptides produced and released in the central and peripheral nervous systems and in a variety of peripheral organs and tissues. PACAP and VIP, which share high amino acidic sequence homology, bind to three different G-protein-coupled receptors, PAC1, VPAC1 and VPAC2, through which they activate signaling cascades to regulate important body metabolic and homeostatic physiological processes. The PACAP and VIP pathways have been linked to the regulation of body weight and fat mass accumulation, and to the development of obesity and metabolic syndrome. In this review article, PACAP and VIP regulation of appetite/satiety, feeding behavior, metabolism, body homeostasis and orexigenic and anorexigenic hormones is discussed.

**Abstract:**

The overwhelming increase in the prevalence of obesity and related disorders in recent years is one of the greatest threats to the global healthcare system since it generates immense healthcare costs. As the prevalence of obesity approaches epidemic proportions, the importance of elucidating the mechanisms regulating appetite, satiety, body metabolism, energy balance and adiposity has garnered significant attention. Currently, gastrointestinal (GI) bariatric surgery remains the only approach capable of achieving successful weight loss. Appetite, satiety, feeding behavior, energy intake and expenditure are regulated by central and peripheral neurohormonal mechanisms that have not been fully elucidated yet. Pituitary Adenylate Cyclase-Activating Polypeptide (PACAP) and Vasoactive Intestinal Polypeptide (VIP) are members of a family of regulatory peptides that are widely distributed in parallel with their specific receptors, VPAC1R, VPAC2R and PAC1R, in the central nervous system (CNS) and in the periphery, such as in the gastrointestinal tract and its associated organs and immune cells. PACAP and VIP have been reported to play an important role in the regulation of body phenotype, metabolism and homeostatic functions. The purpose of this review is to present recent data on the effects of PACAP, VIP, VPAC1R, VPAC2R and PAC1R on the modulation of appetite, satiety, metabolism, calorie intake and fat accumulation, to evaluate their potential use as therapeutic targets for the treatment of obesity and metabolic syndrome.

## 1. Introduction

Neuropeptides are known to be important regulators of digestive and metabolic functions. The control of energy intake and expenditure, glucose homeostasis and body phenotype is achieved through complex interactions between numerous hormones, signaling molecules, neuropeptides and their receptors [1,2,3]. Obesity is a result of a dysregulated balance in the complex molecular interactions that leads to the development of excessive fat accumulation in tissues and organs, and later to the development of metabolic syndrome with its associated disorders, such as diabetes mellitus, hypertension, osteoarthritis, chronic inflammatory diseases, cancer and coronary artery and cerebrovascular diseases [4]. The gastrointestinal (GI) tract plays an important role in the modulation of energy homeostasis by acting as a nutrient sensor in response to luminal stimuli and releasing peptides, functioning as neurotransmitters and hormones [5]. These are considered major regulators of energy intake and expenditure, metabolism, glucose homeostasis and fat accumulation [6]. Vasoactive intestinal polypeptide (VIP) and pituitary adenylate cyclase-activating polypeptide (PACAP) are neuropeptides that belong to the family of gastrointestinal hormones, in addition to secretin, glucagon and GLP-1. VIP and PACAP are widely released in both the central nervous system (CNS) and in the GI tract neurons. Physiologically, VIP and PACAP both play an important role in a variety of gastrointestinal functions, including appetite and food intake regulation, metabolic hormone release, acid secretion, gastric and intestinal motility, sphincter relaxation, neuronal excitability and mucosal inflammatory responses [7,8,9,10,11,12,13].

In this review, the major physiological and metabolic actions of PACAP and VIP through their specific receptors are summarized, and the most recent advances in understanding the role of PACAP and VIP in the pathogenesis of metabolic diseases and obesity disorders are described.

## 2. PACAP

### 2.1. PACAP and Its Receptors

**Figure 1:**
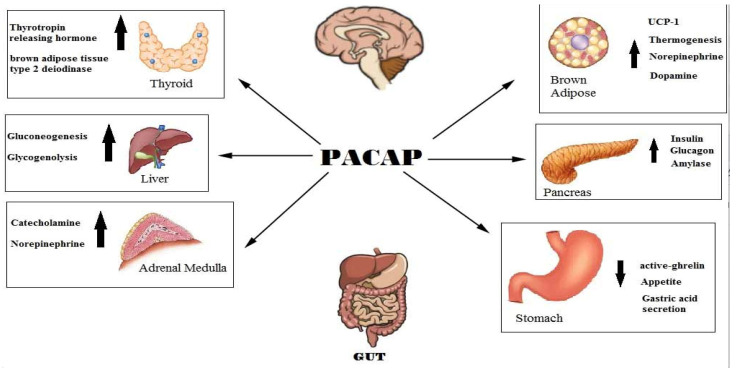
This diagram illustrates the organs, systems and apparatus whose functions are targeted and regulated by the PACAP hormone.

The peptide PACAP, first isolated in 1989 from the pituitary cells of the ovine hypothalamus [14,15], has a highly conserved structure among vertebrate species and belongs to a superfamily of hormones including VIP, gastric inhibitory peptide (GIP), glucagon-like peptide (GLP)-1 and GLP-2, growth hormone-releasing hormone (GH-RH), peptide histidine methionine, peptide histidine isoleucine and exendins. Two different variants of the PACAP hormone are present physiologically, PACAP_1–27_ and the COOH-terminally extended form PACAP_1–38_, which is the most abundant endogenous form [16,17]. The PACAP peptide can activate three different receptors: the PAC1 receptor, known to have 1000-fold higher affinity for PACAP than for VIP, and the VPAC1 and VPAC2 receptors, known to bind to PACAP and VIP with identical affinity [15,18]. PACAP binding to the PAC1 receptor activates both the Gαs and Gq/11 signal transduction pathways, consequently stimulating the adenylyl cyclase and phospholipase C (PLC) pathways, thus resulting in an increase in cAMP and/or inositol phosphate and intracellular Ca^2++^ [19]. PACAP and its high-affinity PAC1 receptor were identified in the central and peripheral nervous systems, in addition to different peripheral organs and tissues [20,21]. PACAP and its receptors are largely expressed in the gastrointestinal tracts of several mammalian species, including humans. PACAP often colocalizes with VIP in neurons and nervous fibers of the submucosal and muscular layers, in the myenteric ganglia of the stomach, in the myenteric and submucous plexuses and in the myenteric ganglia of the esophagus, stomach and small and large intestine [22,23]. Furthermore, the PAC1 receptor was identified on the gastric enterochromaffin-like (ECL) cells of the stomach, in which it regulates the release of histamine and stimulates cellular proliferation [24]. The PAC1 receptor is also expressed in hepatocytes, in which it stimulates glycogenolysis, and in pancreatic exocrine and endocrine cells, in which it promotes the secretion of amylase and the release of glucagon and insulin. PACAP regulates digestive processes by modulating intestinal motility, the relaxation of the lower esophageal sphincter (LES), gallbladder contractility, local blood flow, the secretion of gastric acid, ions, enzymes and hormones, including glucagon and insulin from the pancreas, and immunity. PACAP plays a protective role in the cells of the gut against oxidative stress, inflammation and apoptosis [24,25]. The development of murine genetic models void of PACAP receptor expression has allowed for the identification of many phenotypic and metabolic alterations to suppress food intake, lower body weight and lower plasma levels of insulin [26]. Figure 1 depicts several physiological and pharmacological effects of PACAP.

### 2.2. PACAP’s Central Effects on Appetite and Thermogenesis

PACAP has been shown to play a major role in the central regulation of appetite/satiety, energy balance, metabolism and thermogenesis [27,28,29,30,31,32,33]. Centrally, PACAP has been demonstrated to suppress appetite and feeding through intracerebroventricular injection in vertebrate animals, such as goldfish [29,30], chicks [32,33], rats [28] and mice [29]. In the CNS, PACAP and PAC1 receptor mRNA are highly expressed in the hypothalamus, specifically in the arcuate nucleus (ARC) [34], ventromedial nucleus (VMN) and dorsomedial nuclei [35,36]. Furthermore, PACAP and PAC1 mRNA expression is upregulated in the CNS following excessive feeding [29,37]. In the VMN region of the hypothalamus, which plays a role in enhancing satiety and metabolism through the sympathetic nervous system, PACAP mRNA expression was decreased in fasting conditions, thus suggesting that the PACAP/PAC1 pathway plays a crucial role in the central regulation of appetite/satiety and energy intake via the hypothalamic melanocortin system [38]. Furthermore, PACAP has been shown to play a crucial role in the excitatory drive of the agouti-related peptide-expressing neurons, which control hunger in the ARC of the hypothalamus, an area that is central in the control of hunger and satiety [39]. This enhancement in satiety and suppression of energy intake was replicated using the PAC1-receptor-specific agonist Maxadilan, which also significantly suppressed food intake [40]. Finally, these effects were confirmed pharmacologically using PACAP_6–38_, a PAC1-specific antagonist, which was able to inhibit PACAP’s central effects on food intake [31,38,41,42]. PACAP has also been shown to play a significant role in thermogenesis and body weight regulation [40]. Central injections of PACAP into the VMN or in the posterior region of the stria terminalis bed nucleus produced significant body weight loss 24 hours post-injection [43,44].

### 2.3. PACAP and PAC1’s Peripheral Metabolic Effects

PACAP and PAC1 play a crucial role in the regulation of appetite, satiety and GI function, not only centrally but also peripherally [11]. PACAP and PAC1 have been found to be expressed on gastric nerves [21], and PAC1 is localized also on gastric enterochromaffin-like (ECL) and parietal cells in the gastric mucosa [24,45]. Peripheral administration of PACAP in mice has been shown to inhibit appetite and feeding and to increase satiety, in a dose-dependent manner, for a period of up to 24 hours [11]. The mechanisms underlying these anorexigenic effects of PACAP were suggested to involve the inhibition of active ghrelin release and the modulation of GLP-1, insulin and leptin release, specifically through the PAC1 receptor [11]. Ghrelin is primarily secreted by the PD/1 endocrine cells of the gastric oxyntic mucosa in two different forms: the acyl-ghrelin (active ghrelin), a 28-amino-acid peptide with an *n*-octanoylated serine in position 3, and a des-acylated [des-(Gln14)] ghrelin form [46,47]. Ghrelin peptides have been localized centrally (hypothalamus, pituitary gland) as well as peripherally (pancreas, small intestine, heart, immune system, adipose tissue and in neuroendocrine tumors) [46,47,48]. The main activity of ghrelin is to increase appetite and food intake while regulating energy balance. During fasting, the levels of ghrelin in the blood reach their highest, followed by a decrease in postprandial conditions. In the stomach, PACAP and PAC1 expression has not been detected in the gastric fundus on the PD/1 cells; however, PAC1 receptors have been identified on the ECL cells, which were shown to secrete ghrelin [45,49]. In the stomach, PACAP stimulates PAC1 receptors expressed on enterochromaffin-like (ECL) cells to regulate gastric acid secretion and VPACs’ receptors to trigger somatostatin release from D cells [24,45,50]. Furthermore, PACAP and PAC1 have been shown to modulate hormonal and neural signals that are implicated in metabolism, such as the release of glucagon and insulin from the pancreas [51,52], growth hormone from the pituitary gland, glucocorticoids from the adrenal cortex and catecholamine from the adrenal medulla [53,54]. In the exocrine pancreas, PACAP is a potent stimulator of the acinar release of amylase [55]. PACAP-activating PAC1 receptors on pancreatic β-cells were reported to stimulate insulin secretion [42,43] in both humans and rodents [52,56] through the release of cAMP and through K_ATP_ channels. In addition, PACAP appeared to regulate β-cells’ transcription of insulin, GLUT-1 and hexokinase 1 [57]. Prior publications demonstrated that in PAC1 knockout mice, there was a reduction in insulin responsiveness to meal ingestion, with no increase in postprandial plasma insulin levels, and that the use of the PACAP partial antagonist PACAP_6–38_ blunted the glucose-induced insulin response [11,57]. Furthermore, PACAP was shown to modulate the release of leptin, an anorexigenic hormone synthesized mainly by adipose tissue cells, which is implicated in the regulation of appetite/satiety, body weight and body temperature by acting on hypothalamic neurons [43]. PAC1^−/−^ mice were reported to have abolished fasting and postprandial levels of leptin [11]. In leptin knockout *ob/ob* mice, PACAP mRNA was significantly reduced in fasting conditions, but increased following the intracerebroventricular injection of leptin [43]. Furthermore, the intracerebroventricular injection of leptin in PACAP knockout mice had no significant effect in modulating feeding behavior [58].

### 2.4. Effects of PACAP and PAC1 on Energy Expenditure

The characterization of the roles of PACAP and PAC1 in energy homeostasis has provided insights into the widespread and complex physiological effects of this peptide. PACAP, through its PAC1 receptors expressed in hypothalamic and peripheral sympathetic nervous system fibers, activates the release of sympathetic amines and glucose, and the following uptake of glucose and lipids in the brown adipose tissue with an increase in energy expenditure. Studies utilizing PACAP^−/−^ mice have shown that PACAP is crucial for thermogenesis and thermoregulation, and that PACAP^−/−^ mice have a significantly lower core body temperature, which leads to early postnatal death and altered lipid and carbohydrate metabolism [59,60]. This mechanism was due to the insufficient stimulation of brown adipose tissue by norepinephrine and its precursor dopamine, whose levels were significantly lower in PACAP^−/−^ mice. At 21 °C, PACAP^−/−^ mice were leaner than their littermates due to decreased adiposity; however, this difference was eliminated at 28 °C [59,60,61,62]. This effect was ameliorated by PACAP injection into the VMN, which caused an increase in core body temperature and spontaneous locomotor activity, as well as an increase in brown adipose uncoupling protein 1 mRNA expression [41]. PACAP^−/−^ mice had altered substrate utilization, reduced β3-adrenergic receptor (β3-Ar (Adrb3)) and hormone-sensitive lipase (Hsl (Lipe)) gene expression and increased fibroblast growth factor 2 (Fgf2) gene expression in BAT [61]. Furthermore, in PACAP^−/−^ mice, the thyroid hormone axis was altered, leading to lower mRNA levels of thyrotropin-releasing hormone and brown adipose tissue type 2 deiodinase [62]. PACAP was shown to be expressed in preadipocytes and able to stimulate cAMP production and the phosphorylation of MAPK (ERK1/2) [63]. In primary rat adipocytes, PACAP enhanced lipolysis in the absence of insulin, whereas, in the presence of insulin, it downregulated lipolysis and upregulated lipogenesis. Furthermore, PACAP^−/−^ mice had higher levels of serum cholesterol and triglycerides [59]. PACAP intraperitoneal treatment reduced liver fat accumulation and blocked lipogenesis in a high-fat-diet murine model by activating the FAIM-AMPK-IRb axis to suppress lipogenesis [64]. The central and peripheral regulation of appetite and satiety by PACAP and PAC1 supports the concept that PACAP is an essential sensing and signaling hormone that modulates physiological body energy homeostasis. Therefore, the potential use of PACAP/PAC1 agonists as novel pharmacological agents in the treatment of appetite disorders, obesity and metabolic syndrome is promising and needs further exploration.

## 3. VIP

### 3.1. VIP and Its Receptors (VPAC1 and VPAC2)

**Figure 2:**
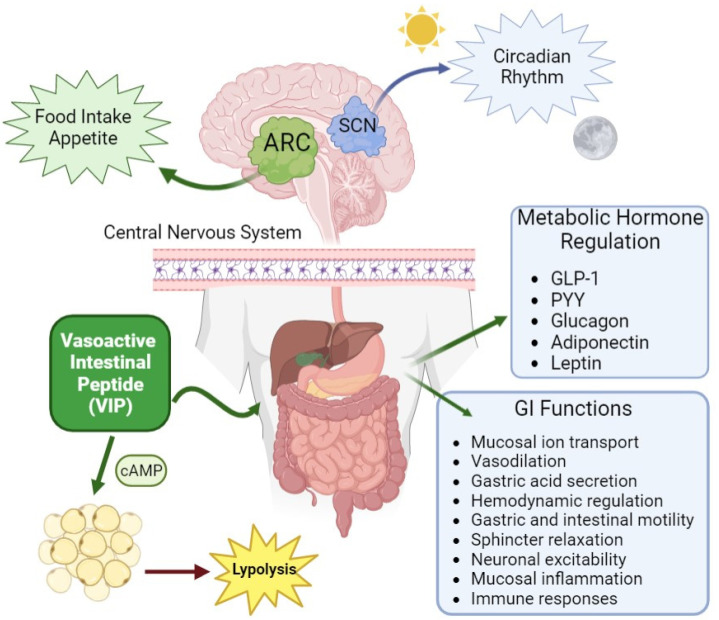
VIP neuropeptide, which is localized in the CNS and in peripheral organs and tissues, regulates, through its specific receptors, important physiological and metabolic functions, such as those illustrated in this figure.

VIP, a highly conserved peptide with 28 amino acids, first isolated from the porcine intestine, specifically binds with equal affinity, as well as PACAP, to two G-protein-coupled receptors, VPAC1 and VPAC2, which activate the adenylate cyclase/cAMP pathway. The different expression of these two VPAC receptors in the CNS, as well as in peripheral organs and tissues, can explain the wide range of physiological effects exerted by this neuropeptide. VIP is a member of the glucagon/secretin peptide family, which includes glucagon, glucagon-like peptide 1 and 2 (GLP-1 and GLP-2) and growth-hormone-releasing factor and gastric inhibitory peptide (GIP) [8,18]. The VPAC1 receptor is largely localized in the central nervous system (cerebral cortex and hippocampus) and peripherally in the liver, intestine, adipocytes, lung and T lymphocytes [65,66]. Similarly, the VPAC2 receptor is localized centrally, with high expression in the thalamus and suprachiasmatic nucleus (SCN) and lower expression in the brainstem, hippocampus, spinal cord and dorsal root ganglia. Peripherally, VPAC2 is localized in the smooth muscle tissue of the cardiovascular, gastrointestinal and reproductive systems [67,68]. VIP, through VPAC1- and VPAC2-specific receptors, plays a major role in several physiological functions and processes, such as the regulation of appetite/satiety, energy intake, feeding rhythms, body mass, metabolism, glucose homeostasis, the circadian pacemaker, intestinal mucosal ion transport, hemodynamic regulation, sphincter relaxation, gastric acid secretion, vasodilatation, neuronal excitability, gastric and intestinal motility and secretion, microbiota and mucosal immunity [8,9,10,12,18,32,33,34,53,69,70,71,72,73,74,75,76] (see Figure 2). In VIP^−/−^ mice, a morphometric analysis of the gastrointestinal tract revealed a reduced intestinal length, a larger cross-sectional diameter of the gut, increased thickness of the muscularis propria and significant mucus accumulation in goblet cells. Furthermore, VIP^−/−^ mice demonstrated delayed gut motility, as shown by the fluorescent dextran method, as well as a 36% decrease in bolus transit [69].

### 3.2. VIP Effects on Appetite, Satiety and Circadian Rhythm

The VIP neuropeptide has a key role in modulating appetite and energy intake. ICV injections of VIP were shown to decrease food intake in different vertebrate animal models, including chicks and goldfish, thus suggesting an anorexigenic role for VIP at the CNS level in the ARC [30,32,33]. VIP^−/−^ mice had altered feeding behavior, showing increased food intake during the 12-h light phase and decreased food intake during the 12-h dark phase, thus demonstrating an altered circadian rhythm of feeding [10]. Overall, in VIP^−/−^ mice, there was a 15% decrease in food intake at the end of a 24-h study period of observation, which could explain the reduced animal body size, slower growth rate and consequent lower metabolic needs. This mechanism could be mediated through the VPAC2 receptor, as feeding behavior observations in VPAC2^−/−^ mice showed a significantly reduced daily amount of food intake [77]. Our group recently demonstrated, by indirect calorimetric analysis, no significant differences in food consumption and the amount of time spent feeding in VPAC1^−/−^ mice, even though there was an increase in the number of feeding bouts during the dark cycle [78]. Previously, other researchers showed that a long-term treatment with a VPAC1 agonist inhibited food intake over a 28-day experimental period [79]. On the other end, Alexander at al. reported that a pretreatment with a VIP antagonist, [Lsy1, Pro2,5, Arg3,4,Tyr6]-VIP, inhibited the food-intake-induced increase in plasma ACTH and corticosterone in fasted rats [80]. In addition, both VIP^−/−^ and VPAC2^−/−^ mice had an altered 3–4 hour advancement in their metabolic and feeding rhythms compared to WT mice [10,77,81]. In VPAC2^−/−^ livers, peripheral clock gene expression was altered at feeding times, even in the absence of a functional SCN clock, thus confirming the role of the VIP/VPAC2 pathway in regulating metabolism [82]. It has been shown that core clock genes are important modulators of body and organ metabolism [83]. An analysis of the light–dark cycle in VIP^−/−^ and VPAC2^−/−^ mice revealed limited alteration of the diurnal rhythms of activity. Furthermore, significant alterations of circadian rhythms were detected in both VIP^−/−^ and VPAC2^−/−^ mice during the dark phase. Activity patterns began 8 h earlier than initially expected based on the shorter previous light cycle free-running period, along with an additional loss of coherence and precision in the circadian locomotor activity rhythm [81,82]. The pacemaker responsible for circadian daily rhythms is localized in the suprachiasmatic nucleus, which expresses both the VIP peptide and VPAC2 receptor and whose pathways are considered responsible for the maintenance of these daily rhythmic functions [82]. Circadian clock genes, which regulate the physiological activities of the gastrointestinal, hepatic, pancreatic, endocrine and renal systems and thermogenesis, have been associated with body homeostasis, metabolism and obesity [83]. Several animals and human studies indicate that the oscillating rhythms of feeding and energy homeostasis are essential in maintaining a healthy body weight, mass composition and metabolism. The genetic lack of expression of the circadian deadenylase gene *Nocturin* caused resistance to diet-induced obesity in mice, which had a lean body phenotype with no reduction in food intake or physical activity and an absence of fatty liver [84]. Our research team has observed similar metabolic and phenotypic characteristics in VIP^−/−^ mice, which, according to a metaGene expression data analysis, had altered absorption of lipids and metabolism, as well as abnormal expression of the PPARγ gene, a fundamental regulator of adipogenesis in adipocytes, and of Srebp-1c, a transcription factor for several lipogenic genes (unpublished data). Similarly, the transcription factor *Bmal1*, which regulates circadian rhythms, is known to modulate adipogenesis and lipid metabolism in mature adipocytes [85]. The VIP pathway genes have been strongly correlated to the development of adiposity and obesity by a genome-wide association analysis of 500,000 SNPs from 1000 United States citizens [86]. In another GWAS study of 89,283 individuals, VIP and other six circadian genes were found significantly associated with morningness, insomnia, depression and BMI [87].

### 3.3. VIP and Its Receptors in Body Composition

VIP is considered an important regulator of development and lipid metabolism [88,89,90]. In rat adipocytes, VIP binding to VPAC2 receptors increased cAMP and lipolysis, with the release of fatty acids and glycerol [91]. Akesson et al. demonstrated that although all PAC1, VPAC1 and VPAC2 receptors are localized in primary rat adipocytes, only VPAC2 promotes lipolysis [92]. VIP is a key body phenotype regulator, significantly enhancing body weight and fat mass accumulation [10]. These data suggest that VIP-mediated pathways can play a significant role in the development of obesity and metabolic syndrome. Our team previously demonstrated, through a 22-week NMR analysis study, that VIP^−/−^ mice had a significant reduction in body fat mass and epididymal fat depots, while maintaining their lean body mass as they aged, with a consequent deficit in fat mass accumulation [10]. Other studies showed that VIP^−/−^ mice, or WT mice treated with a VIP antagonist [88], had significantly lower body weight, slowed development and abnormal social behavior [89,90,91]. The VPAC1 and VPAC2 receptors are expressed in murine preadipocyte NIH3T3-L1 cells and in human and rat adipocytes [92,93]. Thus, VIP signaling through both the VPAC1 and VPAC2 receptors can modulate adipocyte function [94]. In fact, on adipocyte membranes, VIP has been reported to activate adenylate cyclase [93] and to induce lipolysis in primary rat adipocytes by binding VPAC2 receptors [93,95]. In VPAC2^−/−^ male mice, a leaner body phenotype was observed starting at 8 weeks of age, along with lower body weight, length and fat mass as compared to WT controls [95]. The body phenotypes of VPAC2^−/−^ and VIP^−/−^ mice appeared to be similar. VPAC1 receptors were not found to be involved in lipolysis or adipogenesis in vitro [77,93]; however, mice lacking VPAC1 expression presented delayed development and reduced weight [96]. Recently, a 12-week study from our research team [78] showed no significant differences in body weight, fat and lean mass composition in VPAC1^−/−^ mice, thereby demonstrating that the VIP/VPAC1 pathway is not responsible for the phenotype observed in VIP^−/−^ mice [10]. However, we have measured and recorded lower body weights in 8-week-old VPAC1^−/−^ mice and higher mortality in VPAC1^−/−^ pups (unpublished data). Similarly, Fabricious et al. [96] have described developmental delays and lower body weights in VPAC1^−/−^ mice. Another study from Lijnen et al. [97] described that a treatment with monoclonal antibodies to inhibit VPAC1 in WT mice, fed a high-fat diet to induce obesity for 15 weeks, failed to suppress the increase in body weight and fat mass; however, it determined subcutaneous adipocyte hypertrophy. Overall, these findings suggest that the VIP effects on body fat mass accumulation and obesity development are mediated through the VPAC2 pathway.

### 3.4. Effects of VIP on Metabolic Hormone Regulation

VIP is a potent modulator of anorexigenic and orexigenic hormones, through which it can control appetite, energy homeostasis and metabolism. VIP hormone secretion was demonstrated in the rat gastric fundus, therefore suggesting that VIP could potentially modulate the release of other metabolic hormones from the gut mucosa after a meal [98,99]. Plasma VIP concentrations were found elevated after either a carbohydrate meal or water-loading meal [100]. In VIP^−/−^ mice, VIP was shown to regulate fasting and postprandial GLP-1, PYY, adiponectin, glucagon and leptin metabolic hormone plasma levels [10,101]. Furthermore, in VPAC1^−/−^ mice, GLP-1 and PYY plasma levels were significantly more elevated in both fasting and postprandial conditions and glucagon levels were higher only in postprandial conditions, whereas postprandial leptin levels were lower [78]. GLP-1, which is released by intestinal L cells after food intake, can activate glucose-dependent insulin release and pancreatic beta cell growth and suppress glucagon secretion, food intake and gastric emptying. GLP-1 was significantly upregulated in VIP^−/−^ mice and VPAC1^−/−^ mice [10,78]. Therefore, VIP could inhibit GLP-1 through VPAC1. Currently, GLP-1 receptor agonists and dipeptidyl peptidase-4 (DPP-4) inhibitors of GLP-1 degradation have been identified as pharmacological targets for the treatment of type 2 diabetes, as well as to promote satiety and body weight loss [102]. GLP-1 is a potent regulator of glucose homeostasis, increasing insulin and suppressing glucagon secretion in response to blood glucose changes. In our VPAC1^−/−^ study [78], plasma glucagon levels were significantly higher only during postprandial conditions, similarly to our VIP^−/−^ mice observations [10]. This indicates that the VIP/VPAC1 pathway inhibits postprandial glucagon release. Glucagon and insulin, which regulate glycogenolysis and gluconeogenesis, maintain glucose homeostasis. In VIP^−/−^ and VPAC1^−/−^ mice, no significant differences in insulin levels were found in either fasting or postprandial conditions [10,78]. PYY, secreted by the intestinal L cells in the gastrointestinal mucosa, is a satiety signal that reduces food intake. VIP stimulated PYY secretion in isolated perfused rabbit distal colons through cAMP-mediated effects [103]. In our studies, VIP^−/−^ and VPAC1^−/−^ mice had significantly elevated PYY levels, thus showing a very important role for the VIP/VPAC1 pathway in modulating PYY secretion [10,78]. In addition, VIP^−/−^ mice had considerably higher plasma leptin levels compared to WT littermates; thus, leptin could have contributed to their altered feeding behavior and lower body fat content [10,78]. The physiological activation of the VPAC1 receptor pathway could determine the higher postprandial levels of leptin, but not the suppression of leptin release during fasting. VIP would be the mediator of this physiologic mechanism and not PACAP, which was described to mediate the anorexigenic effects of leptin centrally, through specific PAC1 receptor activation, as confirmed by the effects of the PAC1-specific antagonist PACAP_6–38_ [43]. Adiponectin, which is secreted by the adipose tissue and regulates glucose and fatty acid oxidation, was significantly higher in VIP^−/−^ mice [10]. In high-fat-diet-induced obesity models, it was shown that lower levels of adiponectin were closely linked to insulin resistance [104]. Consequently, VIP could play a fundamental role in glucose homeostasis by binding to VPAC2 receptors on pancreatic beta cells to induce insulin release [105,106] and by causing glucagon secretion in a dose-dependent fashion, as described in both normal and diabetic rats [107].

## 4. Conclusions

The regulation of appetite, food intake and metabolism is of major health and social importance given the worldwide increased prevalence of metabolic syndrome, overeating and obesity disorders, which are some of the major causes of morbidity and mortality in human patients. VIP and PACAP both play a significant role in the central and peripheral regulation of appetite/satiety and feeding behavior, energy expenditure, body composition, metabolic hormone regulation and the maintenance of physiological body energy homeostasis. The elucidation and understanding of these pathways that regulate appetite/satiety, metabolism and energy expenditure are critical to our ability to treat obesity disorders. Currently, there is a relative paucity of studies that investigate the role of PACAP and VIP in the treatment of metabolic syndrome. Future studies are needed to further elucidate the receptors and metabolic pathways by which PACAP and VIP regulate energy balance, adipocyte proliferation and fat accumulation. This review demonstrates that the PACAP and VIP pathways are important targets and should be considered in the treatment of overeating and obesity disorders, as well as metabolic syndrome.

## Data Availability

Not applicable.

## Abbreviations

CNS	Central nervous system
GI	Gastrointestinal
GLP-1	Glucagon-like peptide 1
IGF	Insulin-like growth factor
PAC1	Pituitary adenylate cyclase activating polypeptide type 1 receptor
PACAP	Pituitary adenylate cyclase activating polypeptide
PYY	Peptide YY
VIP	Vasoactive intestinal polypeptide
VPAC1	Vasoactive intestinal polypeptide receptor 1
VPAC2	Vasoactive intestinal polypeptide receptor 2 BW body weight
T2D	Type 2 diabetes
GPCR	G-protein-coupled receptor
WT	Wild type
FAIM	Fas apoptotic inhibitory molecule
GIP	Gastric inhibitory peptide
WT	Wild type
HFD	High-fat diet
SREBP	Sterol regulatory element binding protein
AMPK	AMP-activated protein kinase
cAMP	Cyclic adenosine monophosphate
AMPK	AMP-activated protein kinase
cAMP	Cyclic adenosine monophosphate
PKA	Protein kinase A
